# Fatty Acid Metabolites in Rapidly Proliferating Breast Cancer

**DOI:** 10.1371/journal.pone.0063076

**Published:** 2013-05-02

**Authors:** Joseph T. O’Flaherty, Rhonda E. Wooten, Michael P. Samuel, Michael J. Thomas, Edward A. Levine, L. Douglas Case, Steven A. Akman, Iris J. Edwards

**Affiliations:** 1 Department of Internal Medicine, Wake Forest School of Medicine, Winston-Salem, North Carolina, United States of America; 2 Department of Biochemistry, Wake Forest School of Medicine, Winston-Salem, North Carolina, United States of America; 3 Department of Surgical Oncology, Wake Forest School of Medicine, Winston-Salem, North Carolina, United States of America; 4 Department of Public Health Sciences, Wake Forest School of Medicine, Winston-Salem, North Carolina, United States of America; 5 Department of Hematology and Oncology and Cancer Biology, Wake Forest School of Medicine, Winston-Salem, North Carolina, United States of America; 6 Department of Pathology, Wake Forest School of Medicine, Winston-Salem, North Carolina, United States of America; University of Southampton School of Medicine, United Kingdom

## Abstract

**Purpose:**

Breast cancers that over-express a lipoxygenase or cyclooxygenase are associated with poor survival possibly because they overproduce metabolites that alter the cancer’s malignant behaviors. However, these metabolites and behaviors have not been identified. We here identify which metabolites among those that stimulate breast cancer cell proliferation in vitro are associated with rapidly proliferating breast cancer.

**Experimental Design:**

We used selective ion monitoring-mass spectrometry to quantify in the cancer and normal breast tissue of 27 patients metabolites that stimulate (15-, 12-, 5-hydroxy-, and 5-oxo-eicosatetraenoate, 13-hydroxy-octadecaenoate [HODE]) or inhibit (prostaglandin [PG]E_2_ and D_2_) breast cancer cell proliferation. We then related their levels to each cancer’s proliferation rate as defined by its Mib1 score.

**Results:**

13-HODE was the only metabolite strongly, significantly, and positively associated with Mib1 scores. It was similarly associated with aggressive grade and a key component of grade, mitosis, and also trended to be associated with lymph node metastasis. PGE_2_ and PGD_2_ trended to be negatively associated with these markers. No other metabolite in cancer and no metabolite in normal tissue had this profile of associations.

**Conclusions:**

Our data fit a model wherein the overproduction of 13-HODE by 15-lipoxygenase-1 shortens breast cancer survival by stimulating its cells to proliferate and possibly metastasize; no other oxygenase-metabolite pathway, including cyclooxygenase-PGE_2_/D_2_ pathways, uses this specific mechanism to shorten survival.

## Introduction

The growth rate of cancer is commonly estimated by measuring a cell proliferation-related protein, Ki-67, with Mib1 monoclonal antibody. The Mib1 score provides a particularly strong prognostic marker in human breast cancer [1], [2]. However, its relation to this cancer’s content of linoleic acid (LA), arachidonic acid (AA), and the oxygenase-derived metabolites of these two fatty acids (FA) is not clear. Application of LA or AA to breast cancer cell cultures stimulates proliferation apparently because these cells over-express 5-lipoxygenase (LO), 12-LO, 15-LO-1, 15-LO-2, cyclooxygenase (CO)-1 and/or CO-2, and thereby over-produce LA and/or AA metabolites some of which feedback to up-regulate their parent cells’ proliferation. Since dietary excesses of these FA may promote human breast cancer, one or more of these autocoid loops may contribute to this disease [3], [4].

5-LO and 12-LO are over-expressed in the breast cancer of patients who suffer poor survival [5], [6], [7]. Two 5-LO products, 5-hydroxy-eicosatetraenoate (HETE) and 5-oxo-eicosatetraenoate (5-oxo-ETE), and a 12-LO product, 12-HETE, stimulate breast cancer cells in culture to proliferate and may thereby mediate the effect of these enzymes on survival [8], [9], [10], [11], although one study did find 12-LO over expression to be associated with an improved disease-free survival [12]. Low 15-LO-1/15-LO-2 ratios are also associated with shortened breast cancer survival [5], [13]; this suggests that a 15-LO-1 product slows or a 15-LO-2 product speeds the cancer’s growth. Of the metabolites made by these LOs, 15-HETE slows proliferation in several types of cancer cells [14] but its effect on breast cancer cells is unclear, and 13-hydroxy-octadecaenoate (HODE) mediates breast cancer cell proliferation responses to growth factors but its direct effect on these cells also is not clear [4], [14].

Over-expression of CO-2 in breast cancer has been reported to reduce survival [5], [7], [15], [16], not impact survival [12], [15], [16], [17], [18], reduce survival when associated with high Ki-67 levels [19], [20], and improve survival when associated with low Ki-67 levels [20]. In spite of this confusion, CO-2 appears especially important since its pharmacological inhibition reduces the prevalence and progression of this cancer in many, although not all, studies [21], [22], [23], [24]. CO-1 over-expression is associated with increased breast cancer invasiveness [25]; its activity may explain the protective effect in breast cancer of aspirin, which blocks both CO-1 and -2, compared to some disappointing results with drugs that selectively inhibit CO-2 [21]. Aspirin’s targeting of CO-1 may also explain its preventative effects in breast cancers that under-express CO-2 [23]. In any case, both COs metabolize AA to prostaglandin (PG)E_2_ and D_2_, which, perhaps paradoxically, inhibit the proliferation of cultured breast cancer cells [26], [27], [28].

The varying results of the cited studies may reflect an imperfect relation between levels of the oxygenases and their metabolites: an oxygenase’s level does not indicate its activity status, its substrate availability, or the action of other oxygenases which make the same metabolite(s). To clarify the roles of the oxygenases in breast cancer, levels of their metabolites need to be defined and, similar to their parent enzymes, related to factors in the disease that alter survival. Radioimmunoassay studies conducted >20 years ago found that PG-like material in breast cancer was associated with an increase, no change, or a decrease in survival [29] and that PGE-like material was unrelated to tumor grade, estrogen receptors, node metastasis, or survival [30], [31], [32]. Perhaps because of these results and the need for fresh, specially processed tissues for study, newer measurement methods that unambiguously identify the metabolites of the COs and also the LOs in this disease are lacking. We have assayed the growth-promoting activity in human breast cancer cell lines of metabolites whose effects are unknown, measured the metabolites in breast cancer by mass spectrometry (MS)-based selective ion monitoring, and related the findings of these studies to patient Mib1 scores and grade (which includes mitotic index as one of its parameters) as well as node metastasis and other markers of disease severity.

## Materials and Methods

### Subjects

We measured the metabolites in the malignant and normal breast tissue and the fatty acids (FA) in malignant and normal breast tissue, plasma, and RBC of 27 women undergoing surgery for breast cancer at Wake Forest University Medical Center. The study was approved by the Institutional Review Board of Wake Forest University School of Medicine and IRB-approved, written informed consent was obtained from each subject before surgery. Consenting patients were taken in sequence with no knowledge of their disease status; patients with recurrent disease, inflammatory breast cancer, a malignancy other than adenocarcinoma, in situ disease, neo-adjuvant treatment, or failure to have a Mib1 score were excluded retrospectively. All patients included in the study were diagnosed as having invasive ductal carcinoma and were categorized by the following assessments of their cancer: Mib1 score of ≤20 vs. >20 (13 vs. 14 patients, respectively); grade I and II vs. III (Nottingham score [Bloom-Richardson grading system] of 3–5 vs. 6–9; 10 vs. 17 patients) and grade’s component indices, mitosis (2–3 vs. 1; 19 vs. 8 patients), nuclear pleomorphism (3 vs. 2 & 1 19 vs. 8 patients; only 1 patient had a score of 1 [omitting this patient did not effect the statistical significance of results]), and tubule formation (3 vs. 2, 24 vs. 3 patients)(no patient had a tubule index of 1); Her2 negative vs. positive (histological score of 0 or I vs. II or III; 21 vs. 6 patients); estrogen receptor negative vs. positive (histological score of ≤10 vs. >10; 12 vs. 15 patients); progesterone receptor negative vs. positive (histological score of ≤10 vs. >10; 15 vs. 12 patients); triple negative vs. receptor positive for Her2, estrogen, and/or progesterone receptors (12 vs. 15 patients); and tumor size I vs. II–IV (<2 cm vs. ≥2 cm or spread to chest wall or skin; 13 vs. 14 patients). Patients were also categorized based on self-reported race of Caucasian vs. African American (21 vs. 6 patients; no Hispanic, Oriental, or Native Americans occurred in the patient sequence studied); age >50 or ≤50, (7 vs. 20 patients); lymph node metastasis absent (N0) vs. present (N1–N3; 21 vs. 6 patients); and body mass index (BMI) of ≤30 vs. >30 (20 vs. 7 patients).

### Tissue Processing

Breast tissue was removed by lumpectomy or mastectomy and identified visually as malignant (subject to histological confirmation) or normal. Within 15 minutes of removal for lumpectomy or mastectomy specimens, malignant and normal (taken from sites >0.5–1 and >2 cm, respectfully, from malignant tissue) tissues were sampled, placed on ice and dissected into 3 approximately equal-sized specimens (two for metabolites, one for FA). Specimens designated as malignant and normal from the same patient were immediately weighed (wet weight of 3–110 mg for malignant, 5–90 mg for normal) and within 20 min of dissection, separately processed for content of metabolites and FA. For metabolites, sections were put in 1 ml of tris-buffered saline (4°C, pH 7.4) containing 100 µM of diethylenetriaminepentaacetic acid (DTPA), 80 µM of butylated hydroxytoluene (BHT), and 5 ng of the deuterated metabolites listed in Table 1; acidified to pH 3.5; and extracted with hexane:ethyl acetate (1∶1). Extracts were blown dry under a stream of N_2_, taken up in ethanol containing 100 µM of triphenylphosphine (PPh_3_) and 80 µM of BHT, and stored under argon at −80°C. For FA, breast tissue sections were placed in ethanol (4°C) containing 80 µM of BHT and 100 µM of PPh_3_. Whole blood (4 ml) was drawn into ethylenediaminetetracetic acid-containing tubes from patients just before surgery; placed on ice; made 100 µM in DTPA and 80 µM in BHT; and centrifuged (1000 g, 5 min, 4°C) to obtain cell-free plasma and erythrocytes (RBC). RBC were washed in tris-buffered saline (pH 7.4) and suspended in this buffer with 80 µM of BHT and 100 µM of PPh_3_. Extracts and prepared RBC and plasma were stored at −80°C under argon.

**Table 1:** Molecular weights of the precursor and product ions^−1^ of the metabolites and their deuterated internal standards selectively monitored by LC-tandem MS.

	Precursor	Product		Precursor	Product
Analog	ion^−1^m/z	ion^−1^m/z	Analog	ion^−1^m/z	ion^−1^m/z
PGE_2_	351	271	d_7_-5-oxo-ETE	324	210
d_4_-PGE_2_ a	355	275	LTB_4_	335	194.8
PGE_3_	349	269	d_4_-LTB_4_	339.2	196.8
PGD_2_	351	271	15-HETE	319	218.8
d_4_-PGD_2_	355	275	d_8_-15-HETE	327	225.7
5-HETE	319	115	12-HETE	319	179
d_8_-5-HETE^b^	327	115.8	d_8_-12-HETE	327	184
5-HEPE	317	115	13-HODE	295	194.7
5-oxo-ETE	317	203	d_4_-13-HODE	299	197.7

a Internal standard for PGE_2_ and PGE_3._

b Internal standard for 5-HETE and 5-HEPE.

### Metabolite Analysis

Breast tissue extracts were quantified for metabolites by measuring their precursor and parent ions and correcting for processing losses based on the recovery of their deuterated internal standards by liquid chromatography (LC)-tandem MS running in the multiple reaction-monitoring mode. Table 1 lists the metabolites, internal standards, and precursor/product ion m/z values monitored. The MS system was a Waters Quattro II MS with a Z-spray interface automated by a Spark Holland LC and a Reliance Autosampler and Conditioned Stacker maintained at 4°C. We used a cone voltage of 35 V and a capillary voltage of 2.4 kV for HETE, hydroxy-eicosapentaenoate (HEPE), and leukotriene (LT) metabolites and 50 V and 3.5 kV for PG metabolites. The LC system for HETE, HODE, leukotriene (LT), and HEPE metabolites was a Waters Corp YMC ODS-AQ 1 mm I.D.× 100 mm length column eluted at 0.05 ml/min with 2 mM ammonium acetate in H_2_O, pH 8.0, as solvent A and methanol as solvent B in the following gradients: 0 min, 70% B; 0–4 min to 90% B; 4–5 min, 90% B; 5–6 min to 70% B; 6–30 min, 70% B. The LC system for PGs was a Phenomenex Luna Phenylhexyl 1 mm I.D.×150 mm length column eluted at 0.07 ml/min with H_2_O as solvent A and CH_3_CN, 0.1% formic acid, as solvent B in the following gradients: 0–6 min, 20% B; 6–6.1 min to 45% B; 6.1–7.1 min, 45% B; 7.1–7.2 min to 65% B; 7.2–9.2 min, 65% B; 9.2–9.3 min to 20% B; 9.3–15 min, 20% B. We validated this method’s accuracy and precision and determined the linearity of recoveries over 1 pg–100 ng of each metabolite in cultured prostate cancer cells; several repetitions of the response curves gave closely agreeing results and yielded metabolite levels that varied by <15%. The method reliably detected 1 pg of the PG’s and 5 pg of the other metabolites; in general, it showed <15% variations in breast tissue extracts examined in duplicate.

### FA Analyses

Just before analysis, specimens were spiked with an internal FA standard, extracted, ran on gas chromatography, and quantified as described [33]. FA distributions were consolidated into 7 categories: total ω6 FA, AA, LA, total ω3 FA, the ratio of total ω3 to total ω-6 FA (ω3/ω6 ratio), total saturated FA (Sat), and oleate.

### Metabolites and Other Reagents

Deuterated metabolites (Cayman Chemical) and MCF-7 and MDA-MB-231 breast cancer cell lines (American Type Culture Collection (Manassas, VA) were purchased. We prepared 13-HODE and 15-HETE by incubating LA and AA, respectively, with soybean type II LO and 5-oxo-ETE by chemically oxidizing 5-HETE as described [34], [35]. Products of these reactions were purified by reverse- followed by normal-phase high-performance LC; the structure and purity of the products were defined by UV spectroscopy, nuclear magnetic resonance spectroscopy, and MS. The other non-deuterated metabolites were purchased (Cayman Chemical).

### Cell Growth Assays

Cell growth was assayed with Cell Titer96 Aqueous One Solution Cell Proliferation Assays (Promega) as described [10]. Cells, cultured in DMEM with 10% fetal bovine serum, were grown to 50–70% confluency and then challenged with culture medium ± metabolites for 48 hr.

### Data Presentation and Statistical Analyses

Cell growth data are given in OD units at 490 nm for cultures processed in the Promega assay. Metabolite levels are reported as pg/mg of wet tissue weight; FA levels are in µg/mg of wet tissue weight or percentage of total recovered FA. Differences between outcomes observed in normal and malignant tissue were assessed using paired t-tests while differences in outcomes between independent groups (e.g., grade I/II vs. III) were assessed using Student t-tests with the Welch correction used if the equality of variances p-value was <0.05. Correlations between parameters are presented as Pearson coefficients. All probability tests were two-sided with their significances being corrected, where indicated, for multiple observations by the false discovery rate method [36]. Corrected p-values of <0.05 were considered significant.

## Results

### Cell Growth

**Figure 1:**
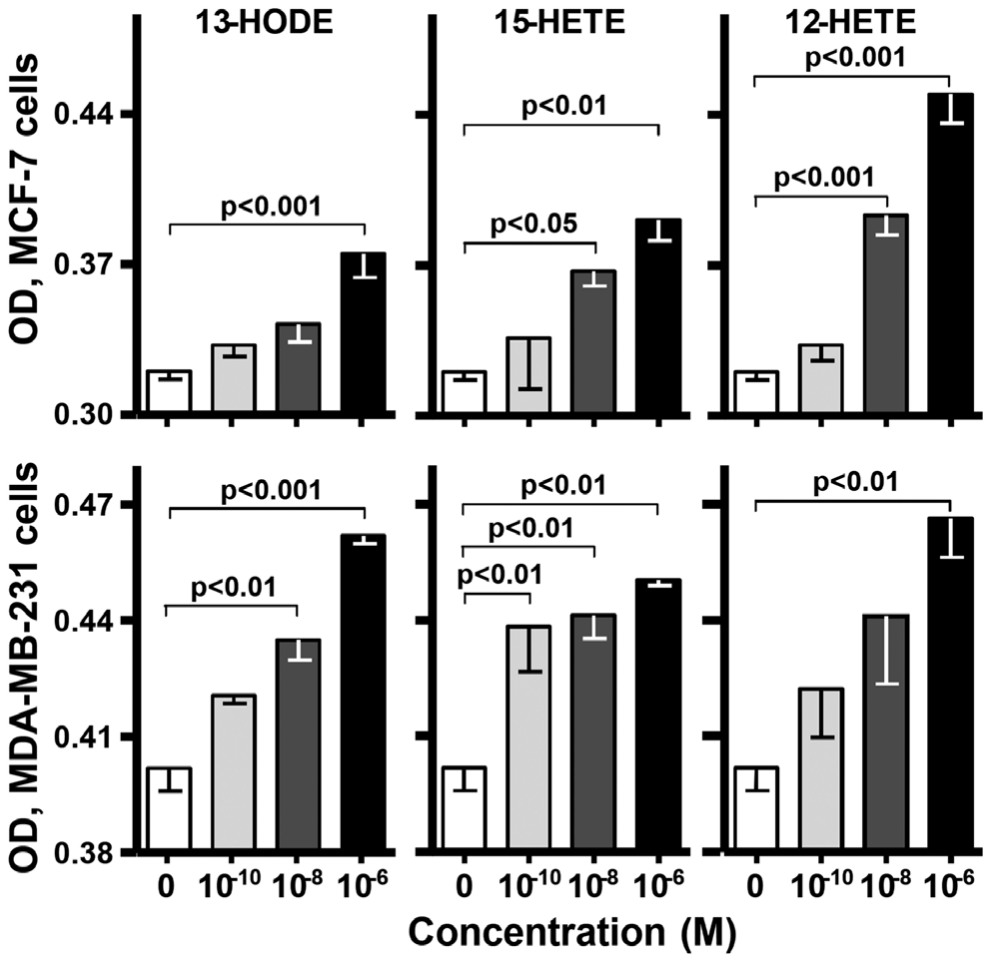
Cell growth. MCF-7 (upper panels) and MDA-MB-231 (lower panels) cell cultures were challenged with a metabolite for 48 hr and assayed for cell density. One-way ANOVA gave the statistical significances shown between comparisons of cells treated with 0 (culture media) or 100 pM–1 µM of the indicated metabolite. Data are means ±SEM in ODU_490_ of 3–6 cultures.

Since the effects of 13-HODE and 15-HETE on growth of breast cancer cells are unclear, we tested them for this. Both metabolites increased cell number in MDA-MB-231 and MCF-7 cultures at ≥100 pM, achieving responses similar to the known [11] effects of 12-HETE (Fig. 1). 5-HETE and 5-oxo-ETE also stimulate these cell lines to proliferate [8], [10] while PGD_2_ and E_2_ exhibit anti-proliferative effects [26], [27], [28].

### Metabolites in Breast Tissue

**Figure 2:**
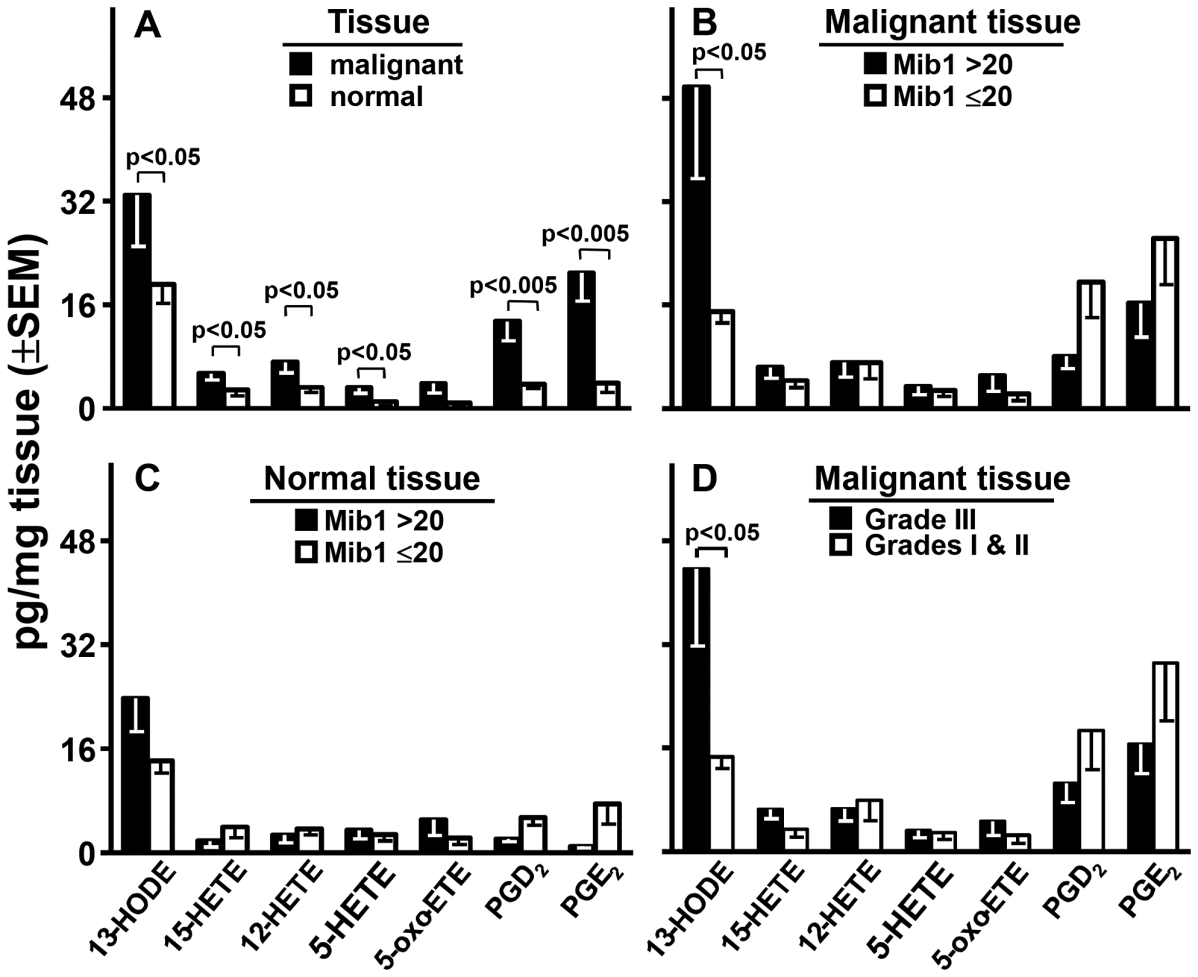
Metabolites and Mib1. Levels of the metabolites are compared by tissue type (panel A), Mib1 score in malignant (panel B) or normal (panel C) tissue; and grade in malignant tissue (panel D). Probability values were defined by paired (panel A) or unpaired (panels B, C, and D) Student t-tests and were corrected for the 7 comparisons made in each panel by the false discovery rate method.

We measured the 10 metabolites and 8 deuterated internal standards listed in Table 1 in malignant and non-malignant breast tissue. PGE_3_ and LTB_4_ were undetectable in all tissues; 5-HEPE was undetectable in 24 malignant and 27 normal tissues. These metabolites were excluded from further analyses. 13-HODE, 15-HETE, 12-HETE, 5-HETE, 5-oxo-ETE, PGD_2_, and PGE_2_ were detected in virtually all malignant and most normal tissues. Their levels, corrected for processing losses by the recoveries of their analogous deuterated internal standards, are given in Fig. 2A. Each metabolite except 5-oxo-ETE was significantly more abundant in malignant than normal tissue with 13-HODE being the dominant metabolite in both tissues.

### Metabolites and Mib1

Mib1 scores of >20 and ≤20 classify breast cancer into respectively faster and slower proliferating diseases with corresponding poorer or better survivals. In breast cancer tissue, 13-HODE stood alone in being significantly related to this classification: it was >3.3-fold higher in patients with >20 Mib1 scores (Fig. 2B). 15-HETE, 5-HETE, and 5-oxo-ETE were slightly higher in tissue with high Mib1 scores; 12-HETE was almost identical regardless of the tissue’s Mib1 score; and PGD_2_ and E_2_ trended 60% and 40% lower, respectively, in tissue with the higher Mib1 scores. In sharp contrast to the findings in cancer tissue, no metabolite was significantly higher in the normal tissue of patients with >20 compared to ≤20 Mib1scores (Fig. 2C). Particularly relevant to this result, the level of 13-HODE in cancer tissue (49.6±14.0 pg/mg, mean ±SEM) was significantly (P<0.05, paired t-test) higher than that of normal tissue (23.8±5.1) in patients with >20 Mib1 scores yet was virtually identical in patients with ≤20 Mib1 scores, i.e. 15.0±1.8 in cancer, 14.2±1.9 in normal tissue.

### Metabolites and Grade

**Figure 3:**
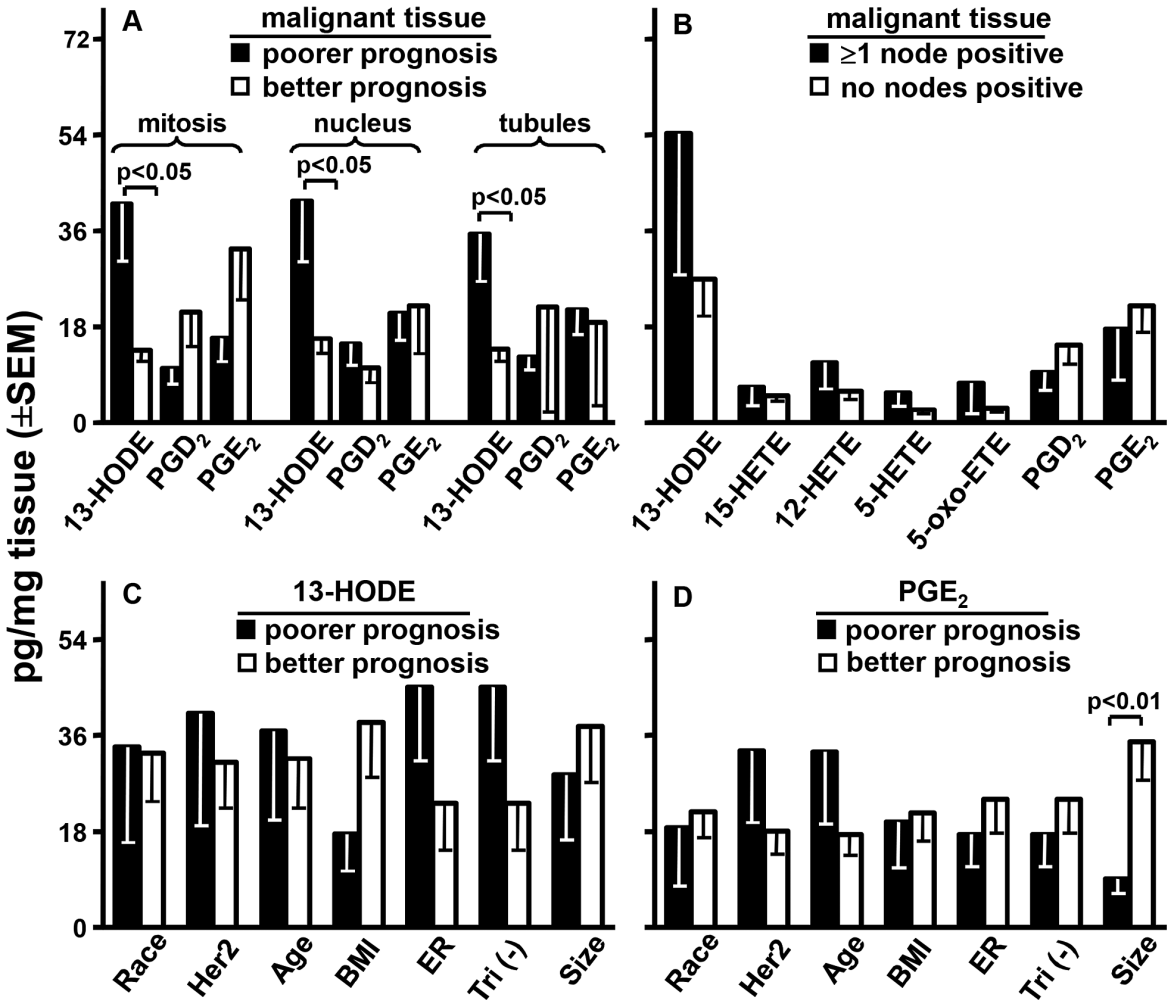
Metabolites and other markers. Malignant tissue levels of the indicated metabolites were compared for poorer or better prognoses by mitosis, nuclear pleomorphism, and tubule formation indices (panel A) or nodal metastasis (panel B). 13-HODE (panel C) and PGE_2_ (panel D) levels were compared by poorer vs. better prognoses for: race, African (closed bars) or Caucasian American (open bars); Her2 score, 2 & 3 (closed bars) or 0 & 1 (open bars); age >50 years (closed bars) or ≤50 years (open bars); body mass index (BMI) >30 (closed bars) or ≤30 (open bars); estrogen receptors (ER) negative (closed bars) or positive (open bars); triple negative (tri (−)) for estrogen, progesterone, & Her2 receptors (closed bars) or not (open bars); tumor size, >2 (closed bars) or ≤ 2 cm (open bars). p Values are from Students t-test corrected for the 3 comparisons in each component of growth (panel A), for the 7 metabolite comparisons (panel B), or for the 7 marker comparisons (panels C and D) by the false discovery rate method. Analysis of these two metabolites for progesterone receptors or for 15-HETE, 12-HETE, 5-HETE, 5-oxo-ETE, and PGD_2_ in all 8 marker categories found no significant differences (data not shown).

Our study population had 1 grade I, 9 grade II, and 17 grade III tumors. Increasing grade predicts increasingly more aggressive disease and poorer survival. We compared grade I and II to grade III (omitting grade I did not alter the significance of this comparison). In malignant tissue, 13-HODE levels were >3-fold higher (p<0.05) and each PG trended lower by >43% in Grade III disease. The other metabolites were at similar levels irrespective of grade (Fig. 2D). 13-HODE’s level in the cancer tissue of patients with grade III disease (44.7±11.8 pg/mg, mean ±SEM; Fig. 2D) was significantly (P<0.05, paired t-test) higher than that in normal tissue (21.6±4.4; not shown); for patients with grade I & II disease, these respective levels were 14.7±1.8 (Fig. 2D) and 15.0±2.1 (not shown). The agreement of grade with Mib1 scores reflected their common basis: grade scores are a combination of 3 proliferation-related indices: mitosis, nuclear pleomorphism, and tubule formation (higher indices for each suggest poorer prognoses). 13-HODE was significantly higher in the cancer (Fig. 3A) but not normal tissue (results not shown) of patients with higher indices for each grade component. PGE_2_ and D_2_ trended lower in the cancer of patients with higher indices for mitosis but not the other two components (Fig. 3A).

### Metabolites and Metastasis

The level of 13-HODE was 2-fold higher in the cancer tissue of patients with ≥1 lymph node positive for disease compared to those with node negative disease (Fig. 3B). This difference only trended toward significance (P = 0.15, Students unpaired t-test) although we stress that there were only 6 node positive patients in our study. 15-, 12-, and 5-HETE and 5-oxo-ETE showed far smaller elevations, and PGD_2_ and E_2_ were respectively 35 and 20% lower, in node positive disease (Fig. 3B).

### Metabolites and Other Markers

We examined 8 other prognostic markers: African or Caucasian American, Her2 receptor presence or absence; age >50 or ≤50 yrs; BMI >30 or ≤ 30; absence or presence of estrogen or progesterone receptors; triple negativity for Her2, estrogen, and progesterone receptors or presence of at least one of these receptors; and tumor size of >2 or ≤2 cm. The first category in each marker carries a poorer prognosis except age which at >50 years is associated with more frequent but not more severe disease. 13-HODE (Fig. 3C), PGE_2_ (Fig. 3D), and PGD_2_, 15-HETE, 12-HETE, 5-HETE, and 5-oxo-ETE (results not shown) levels did not differ significantly between alternate categories of race, Her2, age, BMI, estrogen receptor; triple negative for Her2, and tumor size except PGE_2_ which was significantly (p<0.01) less in larger tumors. In addition, no metabolite showed a significant difference as a function of progesterone receptor (results not shown).

### Metabolite Correlations

In normal breast tissue, 13-HODE levels did not correlate significantly with those of 15-HETE, 12-HETE, PGE_2_, or PGD_2_ (Pearson correlation coefficients of −0.15, 0.31, −0.20, and −0.10, respectively). In cancer tissue, however, 13-HODE was strongly and significantly correlated with 15-HETE (r = 0.63; P<0.01) but not with 12-HETE, PGD_2_, or PGE_2_ (r = 0.49, −0.01, and 0.15, respectively; P values for these correlations are corrected for the 4 observations made in each tissue). Similar result occurred in tissues from patients with >20 Mib1 scores: 13-HODE and 15-HETE levels were significantly correlated in malignant (r = 0.72, P<0.01) but not in normal (r = −0.04) tissue. In sharp contrast to this result, correlations between 13-HODE and 15-HETE in malignant (r = −0.25) and normal (r = 0.18) tissues of patients with ≤20 Mib1 scores were not statistically significant. 13-HODE also failed to correlate significantly with 12-HETE, PGE_2_, or PGD_2_ levels in patients with ≤20 Mib1 scores.

### FA and Mib1

**Figure 4:**
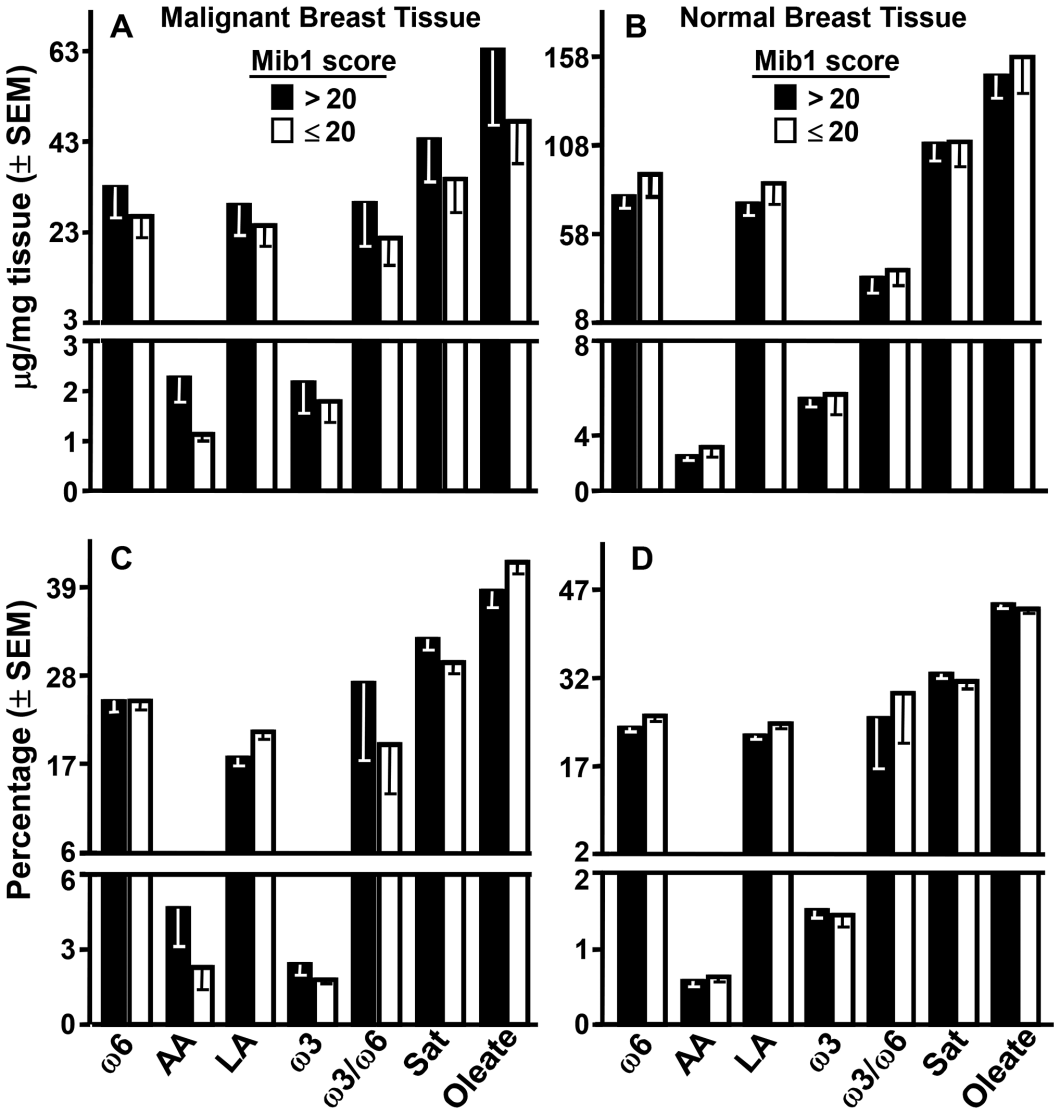
FA and Mib1. Levels of the indicated FA are presented as mass (upper panels) or percentage of total recovered FA (lower panels) in malignant (left panels) and normal (right panels) breast tissue of patients with high or low Mib1 scores. Comparison of the 7 FA parameters on the basis of high or low Mib1 score by Students t-test gave no significant differences even before correction for multiple comparisons; the same analysis in RBC and plasma likewise revealed no significant differences as a function of Mib1 scores (results not shown).

No FA parameter, measured as μg/mg of tissue or percentage of total recovered FA, in malignant (Figs. 4A, 4C) or normal (Figs. 4B and 4D) breast tissue varied significantly as a function of patient Mib1 scores; importantly, this included the precursor to 13-HODE, LA, and the precursor to PGs, AA. A similar lack of relation to Mib1 scores occurred with RBC and plasma from these patients (results not shown).

### FA and Metabolites

There were no significant correlations between the levels of 13-HODE, PGE_2_, or PGD_2_ in malignant tissue and their FA precursors in cancer or normal breast tissue, RBC, or plasma (Table 2). A similar lack of significant correlations occurred in comparing LA and AA levels in these tissues to cancer tissue levels of the 3 metabolites in patients with Mib1>20 scores. Tissue levels of ω6 FA, ω3 FA, total Sat FA, oleate, and ω3/ω6 ratios in the 4 tissues also failed to correlated significantly with cancer tissue levels of the 3 metabolites in all patients or patients with >20 Mib1 scores (results not shown).

**Table 2:** Correlations of LA and AA levels (as masses or percentages of total FA) in malignant breast tissue, normal breast tissue, RBC, and plasma with the levels of 13-HODE, PGD_2_ and PGE_2_ in malignant breast tissue.

	FA mass			FA percentage		
Tissue	13-HODE	PGD_2_	PGE_2_	13-HODE	PGD_2_	PGE_2_
Malignant breast LA	0.02^1^	−0.10	−0.01	−0.20	−0.22	−0.06
Malignant breast AA	0.06	−0.17	−0.17	−0.04	0.11	−0.07
Normal breast LA	−0.22	−0.18	−0.07	−0.32	−0.19	−0.10
Normal breast AA	−0.23	−0.12	−0.16	−0.22	−0.04	0.05
RBC LA	−0.29	−0.18	−0.10	−0.29	−0.19	−0.16
RBC AA	−0.19	−0.13	−0.10	−0.07	−0.05	−0.17
Plasma LA	−0.03	0.04	0.19	−0.09	−0.02	−0.22
Plasma AA	−0.17	0.00	0.02	−0.11	−0.10	−0.24

1 Pearson correlation coefficients between the cited FAs and metabolites. None of the correlations attained statistical significance. There were also no significant correlations between the FA in patients with >20 Mib1 scores (data not shown).

## Discussion

Based on the results in Fig. 1 and the literature (see Introduction), 13-HODE, 15-HETE, 12-HETE, 5-HETE, and 5-oxo-ETE, if impacting proliferation in vivo, would be elevated while PGE_2_ and D_2_ would be reduced in malignant breast tissue with high Mib1 scores. There was evidence for this with 13-HODE: it was the predominant metabolite in breast tissue and its levels were higher in malignant than normal tissue (Fig. 2A) and even higher in malignant tissue from patients with >20 vs. ≤20 Mib1 scores (Fig. 2B). Normal tissue did not show this difference (Fig. 2C). 13-HODE levels were also significantly higher in malignant than normal tissue of patients with >20 but not ≤20 Mib1 scores. No other metabolite presented this pattern. LA levels were not appreciably elevated in malignant breast, normal breast, RBC, or plasma as a function of Mib1 scores (Fig. 4) or significantly correlated with the levels of 13-HODE in malignant tissue (Table 2). Thus, elevated 13-HODE is strongly, positively and, within the range of metabolites tested, uniquely associated with breast cancer proliferation; this does not appear to result from an abundance of its precursor FA. The same pattern of significantly elevated levels of 13-HODE, but not the other metabolites, also associated with the poor prognostic feature of grade (Fig. 2D) and its mitosis, nuclear, and tubular components (Fig. 3A). Neither 13-HODE nor the other metabolites was significantly associated with metastasis (Fig. 3B), race, age, BMI, Her2, estrogen receptor, progesterone receptor, and triple receptor negativity markers (Fig. 3C). Based on these results, 13-HODE appears to fuel proliferation, mitosis, and other components of an aggressive morphology but is less related or unrelated to the remaining markers of severe disease that we tested. There is a proviso here. 13-HODE was far higher, although not significantly, in the cancer of patients with node metastasis (Fig. 3B). Since proliferation markers reflect the potential for, rather than presence of, metastasis, 13-HODE may fuel time-dependent metastases not captured by a single time point study: absent intervention, patients with high Mib1 scores, grade scores, and 13-HODE levels may develop metastasis sooner than those with lower values for these indicators.

15-LO-1 catalyses the oxygenation of AA to 15-HETE and 12-HETE in a 89∶11 ratio, prefers LA over AA as substrate, and makes 13-HODE in excess when both FA are available. This FA preference along with the higher levels of LA compared to AA (Fig. 4) may be responsible for the preferential incremental accumulation of 13-HODE over 15-HETE in rapidly compared to more slowly proliferating cancers (Fig. 2B, 2D, and 3A). Nonetheless, CO-1 and -2 also make 13-HODE [37], [38], [39] and thereby appear to contribute to, for example, the ability of LA to stimulate the growth of MDA-MB-231 cell explants in mice by a CO inhibitor-dependent mechanism [40]. In any event, 15-LO-2 oxygenates AA to 15-HETE but does not make 12-HETE and, like 12-LO and 5-LO, does not attack LA to make 13-HODE [41], [42], [43]. Thus, the effect of CO-1, CO-2, and/or 15-LO-1 on breast cancer survival may reflect their production of 13-HODE. However, we found that: a) 13-HODE and 15-HETE levels were highly and significantly correlated in the cancer but not the normal breast tissue of all patients and patients with >20 Mib1 scores; b) patients with ≤20 Mib1 scores showed none of these cancer tissue findings; and c) no significant correlations occurred between 13-HODE and the other metabolites. This result and the oxygenases’ metabolic profiles argue that the oxygenase capable of making 15-HETE and 13-HODE, 15-LO-1, is the major contributor to 13-HODE overproduction in rapidly proliferating breast cancer. This does not exclude a lesser but still significant role for CO-1/2 in adding to 13-HODE levels in this tissue. Indeed, the beneficial actions of CO inhibitors in breast cancer may reflect such a role. It should also be noted that although human breast cancer cells express all of the relevant oxygenases ([5], [13] and our own unpublished data), the study of breast tissues as a whole does not inform on the cell type originating FA metabolites. An influx of immune cells into a developing tumor may dramatically increase the availability of 13-HODE. The generation of 13-HODE by tissue macrophages is a major feature of late atherosclerotic lesions [44], a mechanism that may extrapolate to malignancies. Analysis of these tissues for the oxygenases by immunohistochemistry may also fail to identify the cells of origin since, as indicated in Introduction, the presence of an oxygenase does not necessarily indicate its metabolite production.

PGE_2_ and D_2_ trended lower in the cancer tissue of patients with Mib1>20 scores (Fig. 2B), grade II & III disease (Fig. 2D), high mitosis rate (Fig. 3A), and node metastasis (Fig. 3B). While these trends failed to attain statistical significance, they did not occur with any other metabolite (Figs. 2B, 2D, and 3A) or marker (except PGE_2_ and tumor size, Fig. 3D). These results are compatible with a notion that reduced levels of PGE_2_ and D_2_ favor breast cancer proliferation.

In conclusion, the metabolites and pathophysiology behind the contributions of FA oxygenases to poor survival in breast cancer has been ill-defined. We find that among the metabolites of the oxygenases known or found here to stimulate breast cancer cell proliferation, 13-HODE stands alone in associating with rapidly proliferating, rapidly dividing, aggressive grade, and perhaps metastasizing breast cancer. Three oxygenases make 13-HODE but correlation studies suggest that its major producer in rapidly proliferating breast cancer is 15-LO-1. Since 15-LO-1 makes other metabolites that are not characterized for proliferative activity in breast cancer cells or measured here, 13-HODE’s contribution to proliferation, division, and metastasis may be complemented or even superseded by other products of 15-LO-1. This caveat also applies to the trends of PGE_2_ and D_2_ to be negatively associated with these parameters of aggressive disease. Nonetheless, our results indicate that 13-HODE is a marker for breast cancer severity and the 15-LO-1/13-HODE pathway is associated with a rapidly proliferating, dividing, and possibly metastasizing phenotype. We propose that the over expression of this pathway speeds breast cancer’s growth and spread. Over expression of the other oxygenase-metabolite pathways, including the CO/PGE_2_/D_2_ pathways, do not use this specific mechanism to worsen the disease.
